# Seroprevalence of *Toxoplasma gondii* in small ruminants from four Caribbean islands

**DOI:** 10.1186/1756-3305-7-449

**Published:** 2014-09-23

**Authors:** Clare M Hamilton, Frank Katzer, Elisabeth A Innes, Patrick J Kelly

**Affiliations:** Moredun Research Institute, Pentlands Science Park, Bush Loan, Edinburgh, EH26 0PZ UK; Ross University School of Veterinary Medicine, PO Box 334, Basseterre, St. Kitts, West Indies

**Keywords:** *Toxoplasma gondii*, Sheep, Goat, Seroprevalence, Caribbean

## Abstract

**Background:**

*Toxoplasma gondii* is a protozoan parasite capable of infecting all warm-blooded animals including livestock. In these animals, the parasite forms cysts in the tissues which may pose a risk to public health if infected meat is consumed undercooked or raw. Little is known of the epidemiology of *T. gondii* in the Caribbean; therefore, the aim of this study was to determine *T. gondii* exposure in small ruminants from four Caribbean island nations.

**Findings:**

Sera from 305 sheep and 442 goats from Dominica, Grenada, Montserrat and St. Kitts and Nevis were examined for *T. gondii* antibodies using an in house ELISA. Reactive antibodies were detected in sheep and goats, respectively, from Dominica (67%, 37/55; 58%, 79/136), Grenada (48%, 40/84; 57%, 54/94), Montserrat (89%, 25/28; 80%, 25/31) and St. Kitts and Nevis (57%, 78/138; 42%, 76/181).

**Conclusions:**

Our results suggest widespread environmental contamination with *T. gondii* oocysts and that small ruminants could be a potentially important source of *T. gondii* infection if their infected meat is consumed undercooked.

## Findings

### Background

*Toxoplasma gondii* is a ubiquitous protozoan parasite capable of infecting all warm-blooded animals, including humans [1]. The definitive host, the cat, passes oocysts in its faeces that contaminate the environment where they can remain viable for long periods of time. Intermediate hosts, such as livestock, are infected by ingesting contaminated water or soil and this results in the formation of tissue cysts, containing *T. gondii,* that can remain viable for the lifetime of the host [2]. Although most infections in small ruminants are asymptomatic there can be abortions, foetal mummification, stillbirths and birth of weak lambs/kids [3]. Humans can become infected with *T. gondii* by ingesting tissue cysts from meat, or oocysts from contaminated food or water. Most infections are asymptomatic; however, there can be severe neurological and pulmonary signs in immunosuppressed people. Infections of a foetus during pregnancy can result in retinochoroiditis and neurological abnormalities [4].

Small ruminants are a very important source of domestic meat production in the Caribbean [5]. Generally, the Caribbean is a net importer of meat, particularly beef and poultry; however, the demand for sheep and goat meat (both referred to as mutton) is high, and any meat produced locally is consumed by the local population and occasionally by foreign tourists interested in tasting local cuisine [5].

The prevalence of *T. gondii* in food animals is higher in pigs, sheep and goats than in cattle [6, 7], and varies worldwide [8]. There are few studies on the prevalence of *T. gondii* in livestock animals in the Caribbean. In Trinidad, a study of animals at slaughter reported *T. gondii* antibodies in 42.9% of goats and 5.5% of pigs [9]. In a more recent study, 23.1% of pigs, 44.1% of sheep, 42.8% of goats and 8.4% of cattle in Grenada and Carriacou were seropositive for *T. gondii* [10].

To provide further data on the epidemiology of *T. gondii* in small ruminants in the Caribbean, we tested sheep and goats from four different islands for reactive antibodies.

## Methods

Sera used in the study had previously been obtained from mixed-breed sheep and goats on the islands of Dominica (15° 25’ North, 61° 20’ West), Grenada (12° 07’ North, 61° 40’ West) Montserrat (16° 45’ North, 62° 12’ West) and St. Kitts and Nevis (17° 20’ North, 62° 45’ West) between 2007 and 2012 and stored at −80°C [11, 12]. All samples were convenience samples collected from healthy, accessible animals on each island, as part of two studies investigating the presence of *Rickettsia africae* [11] and *Ehrlichia ruminantium* [12] in domestic ruminants. The animals were free to graze on open pasture during the day and were housed at night in open-air pens. The climate across the Caribbean is typically tropical with little variation in the coastal areas where the sampling sites were located. Ethical approval for the collection of the blood samples was obtained from the Institutional Animal Care and Use Committee, Ross University School of Veterinary Medicine, St. Kitts. Sera were examined for *T. gondii* antibodies using an in-house ELISA [13], with modifications. In brief, 96-well microtitre plates were coated overnight with 3 μg/ml solubilised RH antigen [14], washed with PBST (PBS with 0.05% Tween-20) and incubated for 2 h at 37°C after addition of 100 μl test or control sera (diluted 1:500 in 1% BSA in PBST) per well. Following washing, 100 μl HRP-conjugated Protein G (diluted 1:20,000 in 1% BSA in PBST) was added to each well and plates incubated for 2 h at 37°C. ELISAs were developed with TMB and reactions stopped with 2 M H_2_SO_4_ before reading ODs at 450 nm. Control sera were pooled samples of 5 sheep experimentally infected with *T. gondii*, and 5 negative control sheep from the same experiment [15]. For each plate, the cut-off value was calculated as two times the percent positivity of the negative control serum relative to the positive control serum (i.e. [2 x (average negative control sera OD/average positive control sera OD)] x average positive control) [16].

## Results

Antibodies to *T. gondii* were detected in sheep and goats, respectively, from Dominica (67%, 37/55; 58%, 79/136), Grenada (48%, 40/84; 57%, 54/94), Montserrat (89%, 25/28; 80%, 25/31) and St. Kitts and Nevis (57%, 78/138; 42%, 76/181) (Table 1).

**Table 1:** **Seroprevalence of** ***Toxoplasma gondii*** **in small ruminants in the Caribbean**

Island	Sheep tested (n)	Sheep positive (n)	% Sheep positive	Goats tested (n)	Goats positive (n)	% Goats positive
Dominica	55	37	67.3	136	79	58.1
Grenada	84	40	47.6	94	54	57.4
Montserrat	28	25	89.3	31	25	80.1
St. Kitts and Nevis	138	78	56.5	181	76	42.0
TOTAL	305	180	59.0	442	234	52.9

## Discussion

Although sample sizes were small, our findings provide the first evidence of *T. gondii* infections in small ruminants from Dominica, Montserrat and St. Kitts and Nevis. Our results, showing that over 40% of sheep and goats from these islands and Grenada were seropositive for *T. gondii,* are consistent with previous studies in the Caribbean that reported seroprevalences of 44% (sheep) and 43% (goats) in Grenada [10], and 43% (goats) in Trinidad [9]. The results are also similar to studies in the USA where seroprevalences vary from 60-74% in sheep [7] and 22-65% in goats [17], and in Central America where seroprevalences of 44% in goats [18] and 38% in sheep [7] have been reported. Similar seroprevalences have also been reported in South America [19]. In our study, the highest seroprevalence was in Montserrat (over 80%); however, the sample size of animals was the lowest of the 4 island-nations and may therefore not be representative of the true seroprevalence. Also, we have no data on the age of the animals sampled, so it is possible that the animals in Montserrat were older than those sampled on the other islands and therefore more likely to be infected, since seropositivity is known to increase with age [20, 21].

With the exception of Grenada, *T. gondii* seroprevalence was higher in sheep than in goats which is a pattern that has previously been reported [10, 20] and may be a result of their foraging behaviour and diet selection. Goats are natural browsers tending to eat leaves and twigs from higher bushes and shrubs, whereas sheep are grazers tending to eat short grasses and clovers close to the soil, thus being more likely to encounter oocysts.

Previous studies in Grenada and St. Kitts and Nevis have reported a high *T. gondii* seroprevalence in cats on the islands, indicating that the environment may be contaminated with oocysts shed in their faeces. A survey of cats in Grenada indicated that 31% of domestic cats, and 28% of feral cats had antibodies to *T. gondii* [22]. Studies in St. Kitts and Nevis have demonstrated that 85% of domestic cats [23] and 74% of feral cats [24] have antibodies to *T. gondii.* Since ingestion of oocysts from contaminated pasture, feed or water is the main route of transmission for herbivores, the high prevalence reported in the present study suggests that the farms on which the animals were sampled had cats on the premises at some point. Furthermore, previous work has demonstrated that 41% of feral chickens in Grenada have antibodies to *T. gondii*, suggesting widespread environmental contamination with oocysts, since chickens are most likely to become infected when feeding on the ground [25].

It is not known whether the animals sampled in this study were destined for the local food chain; however, it is highly likely since there is no exportation of meat from these islands [26] and there is usually insufficient local production of small ruminant meat to cater for local demand [5]. While we cannot say whether the animals were harbouring tissue cysts, the high seroprevalence indicates high levels of exposure to the parasite, and tissue cysts are known to persist for the lifetime of the host [2]. Therefore, there could be a potential public health risk should the meat be consumed raw or undercooked. In the Caribbean, it is generally common practice to overcook or pressure-cook meat which will reduce the risk of transmission of *T. gondii*; however, the high seroprevalence demonstrated in this study indicates high levels of environmental contamination with oocysts, which could potentially offer an alternative route of transmission through contaminated fruit or vegetables [27] or water [28].

Toxoplasmosis is a significant cause of reproductive failure in sheep and goats, leading to economic losses. Although most sheep and goat owners in the Caribbean are small-scale, low-input producers [5], ruminant meat production still serves as a source of income, and any losses due to abortions may have an impact on their livelihood. There is a paucity of data on *T. gondii*-associated abortions in the Caribbean, likely because they are never reported; however, a longitudinal study on a goat herd in Tobago demonstrated increasing *T. gondii* seroprevalence which was significantly associated with increasing abortion rates [29]. If there is widespread environmental contamination with oocysts on the islands in this study, as indicated by the high seroprevalence, reproductive losses due to toxoplasmosis could potentially be a source of economic losses.
